# Incidentalomas discoveries during staging and surveillance for colorectal cancer patients

**DOI:** 10.1097/MD.0000000000045918

**Published:** 2025-11-07

**Authors:** Xu-Rui Liu, Jian Chen, Wei Zhang, Yang Liu, Dong Peng, Dong-Lin Du

**Affiliations:** a Department of Gastrointestinal Surgery, The First Affiliated Hospital of Chongqing Medical University, Chongqing, China; b Department of Radiology, The Second Affiliated Hospital of Chongqing Medical University, Chongqing, China; c Department of Radiology, Qijiang People’s Hospital, Chongqing, China; d Department of Radiology, The First Affiliated Hospital of Chongqing Medical University, Chongqing, China.

**Keywords:** colorectal cancer, computed tomography, incidence, Incidentalomas

## Abstract

This study attempted to evaluate the incidence of incidentalomas based on computed tomography (CT) in colorectal cancer (CRC) patients. CRC patients who obtained plan or enhanced CT for the whole abdominal and pelvis were included at the First Affiliated Hospital of Chongqing Medical University, Chongqing, China. Incidentalomas, including uterine tumors, adrenal gland tumors, renal cancer, pancreatic tumors, prostatic tumors, ovarian tumors, upper-tract urothelial cancer, and gallbladder tumors, were assessed based on all radiology reports by radiologists and surgeons. Moreover, the clinical characteristics of all patients were collected. A total of 7053 CRC patients (mean age, 62.6 ± 12.3; 4139 male) were finally included, 255 (3.6%) patients had an incidentaloma. The proportions of uterine tumors, adrenal gland tumors, renal cancer, pancreatic tumors, and prostatic tumors were 1.3% (92/7053), 1.1% (77/7053), 0.7% (46/7053), 0.1% (15/7053), and 0.1% (10/7053), respectively. Ovarian tumors, upper-tract urothelial cancer, and gallbladder tumors all had an incidence of <0.1%. The prevalence of incidentalomas in CRC patients was 3.6% (255/7053). The most common incidentalomas was uterine tumor, followed by adrenal gland tumor, and renal cancer. These findings highlighted the importance of careful evaluation of abdominal and pelvic CT scans in CRC patients, as timely detection and management of incidentalomas might optimize treatment strategies and improve patient outcomes.

**Key points**

High-resolution computed tomography helps clinicians to assess the incidence of incidentalomas.

Provide some information for the guidelines on the management of colorectal cancer patients with incidentalomas.

## 1. Introduction

In 2020, colorectal cancer (CRC) was the third most common cancer, with an estimated 1.93 million newly diagnosed patients worldwide.^[1]^ In accordance with the National Comprehensive Cancer Network, chest, abdominal, and pelvic computed tomography (CT) is recommended for initial staging.^[2]^ CRC metastatic disease, including liver metastasis and lung metastasis, can also be found in CT imaging. There are 20% of patients have CRC metastatic disease, whose 5-year survival rate is <20%.^[3,4]^ Currently, some other tumors or cancers were detected in staging CT imaging, that caught the attention of doctors.

With the increasing utilization of radiographic imaging, there are more incidentally discovered tumors.^[5–7]^ Adrenal incidentaloma was the most common, with a prevalence of 19% to 35% in ultrasound, and 8% to 65% in autopsy.^[8]^ This was probably because most of the adrenal incidentalomas were benign or clinical-silent.^[9]^ Uterine incidentalomas and renal incidentalomas were also common incidentalomas.^[10,11]^ Uterine fibroid was a common uterine incidentaloma that occurs in 70% of women and could lead to infertility.^[12,13]^ Although abdominal and pelvic CT was widely used for staging and surveillance in CRC patients, the identification of incidentalomas might significantly impact clinical management.^[5–8]^ Some incidental findings might represent synchronous malignancies or benign lesions requiring further workup, potentially influencing treatment strategy, timing of surgery, and patient prognosis. Thus, understanding the prevalence and nature of incidentalomas was essential for guiding follow-up, minimizing unnecessary interventions, and optimizing resource allocation in CRC care.

In CRC patients, some previous studies reported the prevalence and assessed the risk of incidentalomas.^[14–16]^ The clinical factors and management associated with incidentalomas based on CT imaging in CRC patients have not been well assessed. The purpose of this study was to provide more evidence on incidentalomas detected during routine abdominal and pelvic CT in CRC patients, with the intent to support future clinical awareness and inform discussions on incidentaloma evaluation strategies in oncologic imaging.

## 2. Materials and methods

This retrospective observational study was conducted at the First Affiliated Hospital of Chongqing Medical University, Chongqing, China from Jan 2011 to Dec 2021. Ethical approval was obtained from the institutional review board of the First Affiliated Hospital of Chongqing Medical University (2022-134-2). Informed consent was received from all patients. This single-center study was based on a secondary analysis of existing clinical records and imaging data. No additional patient contact or clinical intervention was involved.

### 2.1. Patients

Patients with newly diagnosed CRC were included (n = 8152). The exclusion criteria were as follows: 1. patients who were initially diagnosed with recurrent CRC (n = 47); and 2. Incomplete CT imaging could not lead to under-detection of incidentalomas (n = 1052).

### 2.2. CT protocol

All patients had at least one plan or contrast-enhanced CT scan of whole abdominal and pelvic within 3 months before any CRC treatments. This hospital used automatic dose modulation for taking enhanced CT. According to guidelines, a 10-mm threshold was applied for lesion evaluation. Radiology reports were reviewed to identify incidentalomas involving the uterus, adrenal glands, kidneys, pancreas, prostate, ovaries, gallbladder, and upper urinary tract. All CT images were initially evaluated by 2 professional radiologists. As for patients with suspected incidentalomas, CT imaging was reevaluated by a surgeon. The judgment was positive rather than negative when faced with an equivocal case.

### 2.3. Definition

Incidentalomas were defined as any tumors or cancers found in CT except for lung and liver cancer, because of the high CRC metastasis probability of lung and liver cancer. The tumor node metastasis (TNM) stage of CRC was identified according to the 8th edition guideline of the American Joint Committee on Cancer.^[17]^

### 2.4. Data collection

This study was based on data obtained from the institutional electronic medical record system, radiology information system, and picture archiving and communication system of the First Affiliated Hospital of Chongqing Medical University. Clinical characteristics included age, sex, body mass index, history of smoking, history of drinking, comorbid hypertension, comorbid type 2 diabetes mellitus, comorbid coronary heart disease, history of abdominal history, tumor location, tumor size, TNM stage, preoperative hemoglobin, and preoperative albumin. The clinical information was collected from the electronic medical record, while imaging data were obtained from picture archiving and communication system and radiology information system databases. These included both plain and enhanced abdominal-pelvic CT scans performed as part of routine staging or surveillance.

### 2.5. Statistical analysis

Continuous variables were expressed as mean ± standard deviation, and frequency variables were expressed as n (%). SPSS (version 22.0) software was used for data analysis.

## 3. Results

### 3.1. Patients

**Figure 1.**
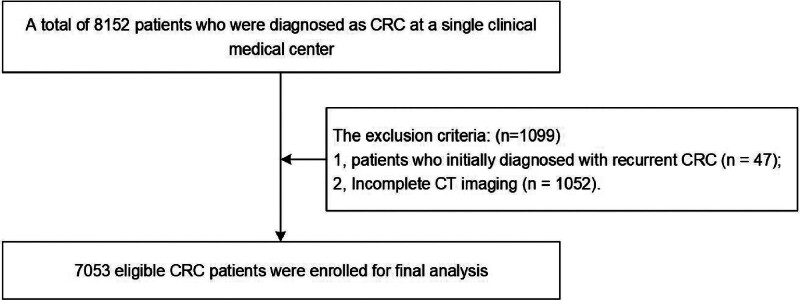
Flow chart of patient selection.

Finally, a total of 7053 patients were included in this study. The selection process and inclusion and exclusion criteria are shown in Figure 1.

### 3.2. Incidence of incidentalomas

**Figure 2.**
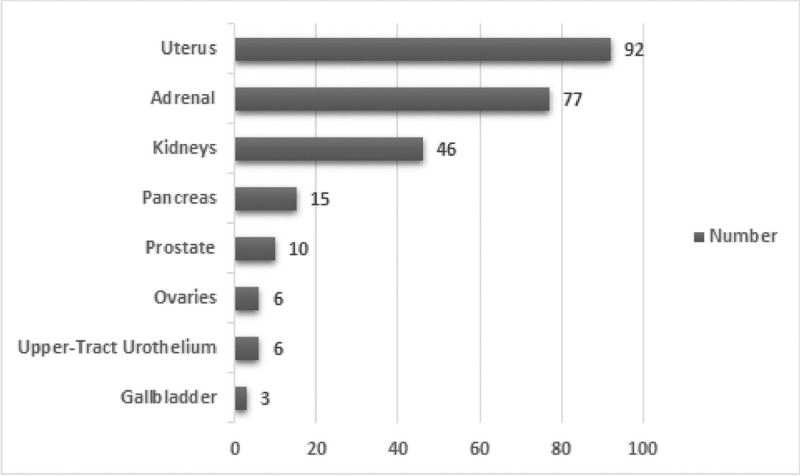
Incidence of incidentalomas.

Incidentaloma occurred in 255 (3.6%) CRC patients. Uterine tumors, adrenal tumors, and kidney cancer were the 3 most common incidentalomas, together accounting for 84.3% of the total amounts. Uterus tumors, adrenal gland tumors, renal cancer, pancreatic tumors, prostatic tumors, ovarian tumors, upper-tract urothelial cancer, and gallbladder tumors were discovered in 92, 77, 46, 15, 10, 6, 6, and 3 patients, respectively (Fig. 2). Notably, all incidentalomas identified were solitary findings; no patients were found to have more than one incidentaloma.

### 3.3. Clinical characteristics

Of the 7053 eligible CRC patients, 4139 (58.7%) were males, and 2914 (41.3%) were females. 2650 (37.6%) patients had a history of smoking, and 2140 (30.3%) patients had a history of drinking. As for comorbid disease, 1767 (25.1%) patients were with hypertension, 774 patients were with type 2 diabetes mellitus (11.0%), and 300 patients were with coronary heart disease (4.3%). 3278 (46.5%) were diagnosed with colon cancer, and 3775 (53.5%) were diagnosed with rectal cancer. 1342 (19.0%), 2844 (40.3%), 2515 (35.7%), and 352 (5.0%) were classified as TNM stage I, II, III, and IV CRC. More clinical characteristics, including age, body mass index, abdominal surgery history, tumor size, hemoglobin level, and albumin level were in Table 1.

**Table 1:** Clinical characteristics of CRC patients.

Characteristics	No. 7053
Age, yr	62.6 ± 12.3
Sex	
Male	4139 (58.7%)
Female	2914 (41.3%)
BMI, kg/m^2^	22.6 ± 3.2
Smoking	2650 (37.6%)
Drinking	2140 (30.3%)
Hypertension	1767 (25.1%)
T2DM	774 (11.0%)
CHD	300 (4.3%)
Abdominal surgical history	1721 (24.4%)
Tumor location	
Colon	3278 (46.5%)
Rectum	3775 (53.5%)
Tumor size	
< 5 cm	4234 (60.0%)
≥ 5 cm	2819 (40.0%)
TNM stage	
I	1342 (19.0%)
II	2844 (40.3%)
III	2515 (35.7%)
IV	352 (5.0%)
Hemoglobin, g/L	121.3 ± 24.0
Albumin, g/L	39.9 ± 5.5

Variables are expressed as the mean ± SD, n (%).

BMI = body mass index, CHD = coronary heart disease, T2DM = type 2 diabetes mellitus.

### 3.4. CRC patients with incidentalomas

**Figure 3.**
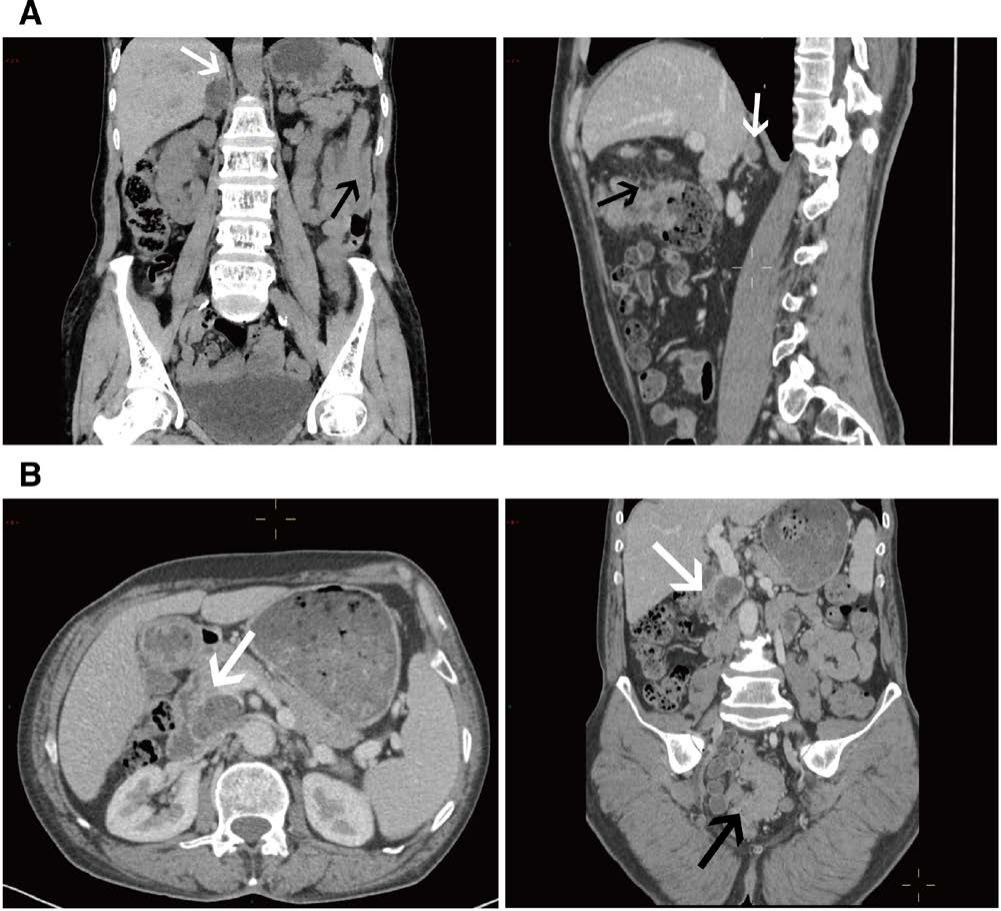
(A) Adrenal incidentalomas. Two patients with colon cancer (black arrows) and adrenal nodule (white arrows) in the right adrenal gland. (B) Pancreas incidentalomas. A 61-year-old man with 5.5 cm rectal cancer (black arrow) and a 2.2 × 3.8 cm irregular low-density mass (white arrows) of pancreas on transverse and coronal CT images.

**Figure 4.**
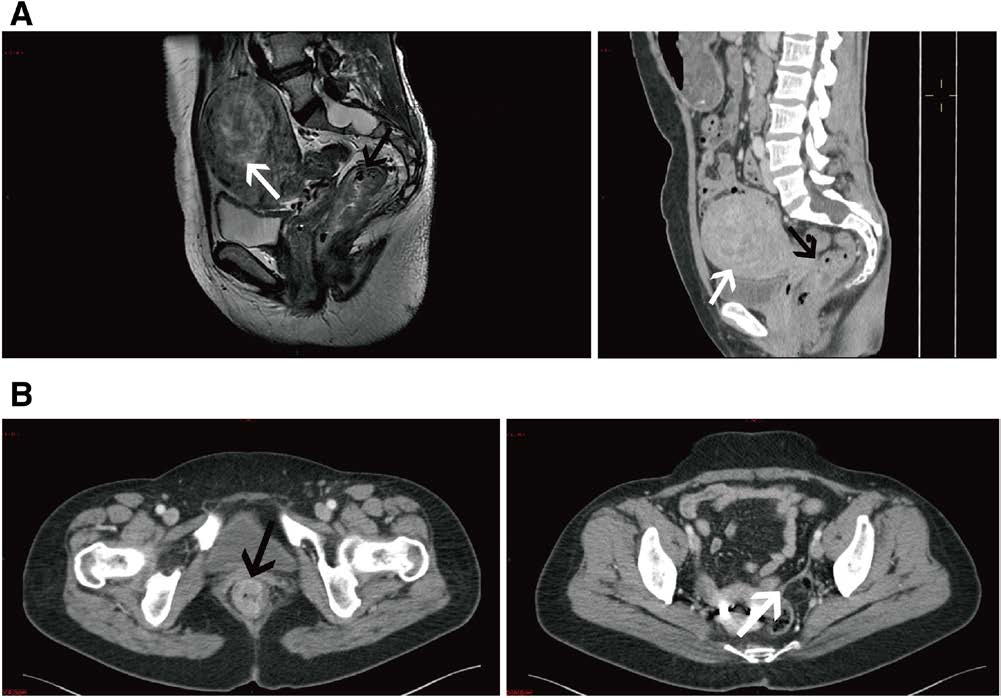
Pelvic incidentalomas included uterus and ovary incidentalomas. (A) A 52-year-old woman with rectal cancer (black arrow) and a 6.7 cm uterus fibroid (white arrows) on T2WI median sagittal MR and CT images. (B) A 50-year-old woman with rectal cancer (black arrow) and a 2.5 × 2 cm teratoma (white arrows) in the left ovary.

**Figure 5.**
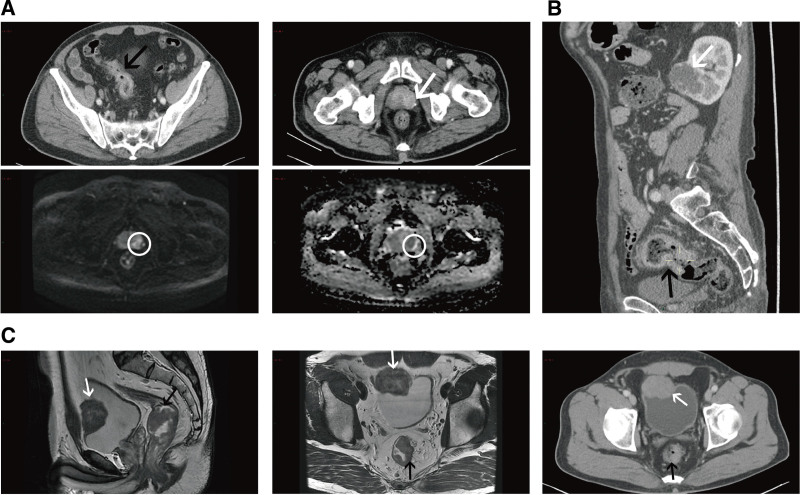
Urinary system incidentalomas included prostate, renal and bladder incidentalomas. (A) A 77-year-old man with sigmoid cancer (black arrow) and abnormal density shadow in the left peripheral zone of the prostate (white arrow and white circles) on CT and MR images. (B) A 73-year-old man with 5 cm sigmoid cancer (black arrow) and a 4.3 × 3.7 cm uneven-density mass (white arrows) in left kidney. (C) A 52-year-old man with rectal cancer (black arrow) and a 4.7 × 2.7 × 3.9 cm mass (white arrows) in bladder on median sagittal and transverse CT images.

The CT and magnetic resonance imaging images of patients with incidentalomas were shown in Figures 3–5. Figure 3A showed 2 patients with colon cancer and right adrenal incidentalomas. Figure 3B showed an irregular low-density mass in the pancreas of a patient with rectal cancer. As for pelvic incidentalomas, uterus and ovary incidentalomas were shown in Figure 4. Figure 4A included a CT and a T2WI median sagittal images, that showed a 6.7-cm uterus fibroid in a rectal cancer patient. And Figure 4B was a rectal cancer patient with left ovary teratoma. In addition, Figure 5 shows urinary system incidentalomas. Figure 5A included enhanced CT and magnetic resonance imaging images, that were taken from a 77-year-old man with sigmoid cancer and prostate cancer. Figure 5C showed a bladder incidentaloma in a rectal cancer patient based on enhanced median sagittal and transverse CT images. Figure 5B was a sigmoid cancer patient with incidental kidney cancer.

## 4. Discussion

This study enrolled 7053 patients diagnosed with CRC. Incidentalomas were investigated in 255 (3.6%) patients. Uterine incidentalomas, adrenal incidentalomas, and renal incidentalomas were the most prevalent incidentalomas, which together accounted for 84.3% of the total amounts.

Incidentaloma could be discovered during staging and surveillance imaging for many cancers, including pancreatic cancer, gastric cancer, and liver cancer.^[18–20]^ Since surgery was the cornerstone of treatment for many cancers, there were also some incidentalomas discovered during surgeries.^[21,22]^ In our study, liver and lung tumors were excluded from incidentalomas because of the high potential of CRC metastasis. Other tumors found in abdominal CT images, including uterine tumors, adrenal gland tumors, renal cancer, pancreatic tumors, prostatic tumors, ovarian tumors, upper-tract urothelial cancer, and gallbladder tumors, were less likely to metastasize from CRC.

As for the mechanisms of CRC development, there are 3 important signaling pathways, including the mitogen-activated protein kinase pathway, the p53 pathway, and the transforming growth factor-beta pathway.^[23,24]^ In invasive CRC, shed cancer cells can enter the circulation. When disseminated cancer cells enter the portal circulation, they would further transport to the liver sinusoids and liver parenchyma. Cancer cells could also infiltrate the lung parenchyma through general circulation.^[25,26]^

Besides the metastasis sites of liver and lung metastases, a small percentage of CRC will metastasize to some rare sites including ovarian, uterus, biliary, and bladder.^[27–30]^ The incidence of these metastasis diseases is relatively low, with an incidence of <5%. However, the infiltration of CRC cells to other organs is rarely studied and may involve invasion mechanisms of other tumors.^[31–33]^ Therefore, we defined these other tumors as incidentalomas rather than metastatic tumors.

Currently, the guidelines for the management of incidentalomas were relatively lacking, but the occurrence of incidentalomas might have an impact on cancer treatments.^[10,34–36]^ Adrenal gland tumor was the most common incidentalomas and received some attention.^[37]^ Patients with adrenal incidentalomas were recommended to receive dexamethasone suppression tests and regular follow-ups, other appropriate biochemical and imaging were recommended according to individualized needs.

However, adherence was poor, especially in some primary hospitals.^[38]^ The malignancy and mortality rates of incidental adrenal tumors were relatively low, and the symptoms were not life-altering, which might account for the low adherence of patients with adrenal incidentalomas.^[39,40]^ Doctors need to weigh the cost of the additional tests for detecting malignancy incidentalomas. Moreover, all incidentalomas deserve attention, and guidelines need to be considered with economic effects to avoid futile medical interventions.

This study was the first study to assess the incidence of all abdominal incidentalomas in CRC patients, and the study was based on a large sample dataset. All images were evaluated by professional radiologists and surgeons to ensure their accuracy. However, the current study was conducted at a single center in southwest China. As for patients with incidentalomas, pathologic confirmations were a lack in the majority of patients, which limited the analysis of the malignancy rate of incidentalomas. Therefore, further studies conducted in multicenter institutions are needed to clarify the interrelationship between incidentalomas and cancers.

In conclusion, the prevalence of incidentalomas in CRC patients was 3.6% (255/7053). The most common incidentalomas was uterine tumor, followed by adrenal gland tumors, and renal cancer. Our findings might provide supportive data for future evaluations regarding the management of incidentalomas detected during routine oncologic imaging, particularly in CRC populations.

## Acknowledgments

We acknowledged all the authors in this article.

## Author contributions

**Conceptualization:** Dong Peng.

**Data curation:** Jian Chen, Yang Liu.

**Methodology:** Dong Peng.

**Resources:** Jian Chen.

**Software:** Xu-Rui Liu.

**Supervision:** Wei Zhang, Dong-Lin Du.

**Validation:** Dong-Lin Du.

**Writing – original draft:** Xu-Rui Liu.

**Writing – review & editing:** Dong Peng, Dong-Lin Du.
